# Association between preprocedural information and anxiety in patients undergoing gastrointestinal endoscopy with sedation: a prospective observational study

**DOI:** 10.3389/fmed.2026.1851863

**Published:** 2026-08-27

**Authors:** Roberto Quirós-Zamorano, Raquel Romero-Tejedor, Miriam Álvarez-Villarreal, Francisco Javier García-Sánchez

**Affiliations:** 1 Outpatient Unit, Instituto de Investigación Sanitaria Hospital Puerta de Hierro Segovia Arana (IDIPHISA), Hospital Universitario Infanta Cristina, Parla, Spain; 2 Research Nursing Unit, Instituto de Investigación Sanitaria Hospital Puerta de Hierro Segovia Arana (IDIPHISA), Hospital Universitario Infanta Cristina, Parla, Spain; 3 Emergency Room and Surgical Prehabilitation Unit, Instituto de Investigación Sanitaria Hospital Puerta de Hierro Segovia Arana (IDIPHISA), Hospital Universitario Infanta Cristina, Parla, Spain; 4 Department of Medicine, Universidad Complutense de Madrid (UCM), Madrid, Spain

**Keywords:** gastrointestinal endoscopy, patient education, patient-centered care, preprocedural anxiety, sedation

## Abstract

**Background:**

Preprocedural anxiety is common among patients undergoing gastrointestinal endoscopy and may be influenced by the quality of information provided before the procedure. However, real-world evidence on the relationship between information delivery and anxiety levels remains limited.

**Methods:**

A prospective observational cross-sectional study was conducted in the endoscopy unit of a tertiary hospital between June and September 2024. A total of 351 adult patients scheduled for elective gastroscopy or colonoscopy with sedation were consecutively included. Preprocedural anxiety and information needs were assessed using the Amsterdam Preoperative Anxiety and Information Scale (APAIS). Sociodemographic variables, referral pathways, and the adequacy of preprocedural information were also analyzed. Prevalence ratios (PR) with 95% confidence intervals were estimated using modified Poisson regression models.

**Results:**

Clinically significant anxiety (APAIS anxiety subscale ≥11) was observed in 30.2% of patients, and high information needs (APAIS information subscale ≥8) in 34.0%. Women showed significantly higher anxiety levels than men (*p* < 0.001). Lack of explanation about the procedure was the only variable significantly associated with higher anxiety (PR 1.61; 95% CI 1.14–2.25; *p* = 0.0074). No significant differences in anxiety were found according to the healthcare professional providing information (*p* = 0.220).

**Conclusion:**

Nearly one in three patients undergoing gastrointestinal endoscopy with sedation experiences clinically significant preprocedural anxiety, with women and inadequately informed patients constituting the highest-risk groups. These findings support the implementation of standardized, patient-centered educational strategies in endoscopy units.

## 1 Introduction

Gastrointestinal endoscopy, including gastroscopy and colonoscopy, is a widely used diagnostic and therapeutic procedure in clinical practice, playing a central role in the management of a broad range of digestive diseases (1, 2). The incorporation of sedation has significantly improved patient comfort, reduced procedural pain, and enhanced both patient and clinician satisfaction, making it a standard component of modern endoscopic practice (3, 4).

Despite these advances, preprocedural anxiety remains a common and clinically relevant issue. Anxiety is defined as a transient emotional state characterized by feelings of tension, apprehension, and increased autonomic activity, which may arise in response to perceived threats or uncertainty (5). In the context of gastrointestinal endoscopy, anxiety may be triggered by multiple factors, including fear of discomfort, concerns about sedation, uncertainty regarding potential diagnoses, and lack of understanding of the procedure (6, 7). Notably, anxiety tends to peak immediately before entering the endoscopy room, potentially affecting patient cooperation and procedural tolerance (8).

The presence of preprocedural anxiety has important clinical implications. Elevated anxiety levels have been associated with poorer patient experiences, increased sedative requirements, and reduced tolerance to endoscopic procedures (9, 10). Therefore, identifying modifiable factors that contribute to anxiety is essential to improve both patient-centered outcomes and procedural quality.

Among these factors, the adequacy of preprocedural information has emerged as a key determinant. Providing clear, structured, and patient-centered information may reduce uncertainty, improve understanding, and alleviate anxiety (11, 12). However, in real-world clinical settings, the information provided to patients is often heterogeneous, varying in content, depth, and delivery depending on the healthcare professional and the care setting, such as primary care or hospital-based services.

Although previous studies have explored anxiety in endoscopic procedures, there is limited evidence regarding how the quality and completeness of routinely delivered information influence anxiety levels and information needs in clinical practice. In addition, the potential differences associated with the source of information—whether provided by primary care physicians or hospital specialists—remain insufficiently understood.

The Amsterdam Preoperative Anxiety and Information Scale (APAIS) is a validated and widely used instrument that allows for the simultaneous assessment of anxiety levels and information needs in preprocedural settings, providing a clinically relevant approach to this issue (13).

In this context, the aim of this study was to evaluate preprocedural anxiety levels and information needs in patients undergoing gastrointestinal endoscopy with sedation and to analyze their association with the adequacy and source of the information received. Additionally, differences according to sex and age were explored to identify potential high-risk groups and inform targeted interventions.

## 2 Materials and methods

### 2.1 Study design and settings

A prospective observational cross-sectional study was conducted in the endoscopy unit of Hospital Universitario Infanta Cristina (Parla, Madrid, Spain) between June 1 and September 30, 2024. This is a high-volume tertiary referral center performing approximately 4,778 endoscopic procedures per year, encompassing standard procedures (gastroscopy, colonoscopy, and rectoscopy) and advanced interventions [endoscopic retrograde cholangiopancreatography (ERCP) and endoscopic ultrasound]. Elective gastroscopy and colonoscopy with sedation—administered either by a dedicated anesthesiologist or by the endoscopist—represent the predominant procedure categories and constituted the eligible population for this study.

Ethical approval was obtained from the Research Ethics Committee of Hospital Universitario Puerta de Hierro Majadahonda (Protocol PI 166/24). The study was conducted in accordance with the Declaration of Helsinki.

### 2.2 Participants

Adult patients (≥18 years) scheduled for elective gastroscopy or colonoscopy with sedation were consecutively recruited. Exclusion criteria included: (1) emergency procedures without prior patient information; (2) sedation administered before questionnaire completion; and (3) inability to complete the questionnaire due to cognitive impairment, language barrier, or any other cause.

The sample size was estimated using the formula for proportions with a 95% confidence level (*Z* = 1.96), a precision of 5%, and a conservative expected prevalence of anxiety of 50%. Applying a finite population correction based on the estimated annual volume of the unit (*N* = 4, 778 procedures), this yielded a minimum required sample of 356 patients. A target of 350 patients was set for practical reasons, representing a conservative approximation of this estimate.

### 2.3 Data collection

Participants completed a structured questionnaire prior to the procedure after providing written informed consent. Data collected included:

- Sociodemographic variables: age (years) and sex
- Type of endoscopic procedure (gastroscopy or colonoscopy)
- Referral source (primary care physician or hospital specialist)
- Healthcare professional providing preprocedural information (primary care or hospital-based)
- Adequacy of preprocedural information across six predefined domains: (1) explanation of the procedure, (2) explanation of its effects, (3) explanation of procedural risks, (4) explanation of sedation, (5) explanation of sedation risks, and (6) preparation instructions

Patients were considered adequately informed if they reported receiving information on all six predefined aspects of the procedure.

Preprocedural anxiety and information needs were assessed using the validated Spanish version of the APAIS (13). A score ≥11 on the anxiety subscale (items 1, 2, 4, 5; maximum 20) was considered indicative of clinically significant anxiety. A score ≥8 on the information subscale (items 3, 6; maximum 10) was considered indicative of high information needs.

### 2.4 Statistical analysis

Data were managed using REDCap and analyzed with R software (version 4.5.2; R Foundation, Vienna, Austria). Continuous variables were expressed as mean ± standard deviation, and categorical variables as frequencies and percentages.

Group comparisons for continuous variables were performed using Student's *t*-test, and chi-square test was used for categorical variables. Statistical significance was set at *p* < 0.05. (two-tailed).

To evaluate the association between the adequacy of preprocedural information and clinically significant anxiety (APAIS anxiety subscale ≥11), modified Poisson regression models with robust variance estimation were fitted for each of the six informational domains. Results are reported as prevalence ratios (PR) with 95% confidence intervals (CI). All models were unadjusted given the exploratory nature of the analysis and the absence of a predefined set of confounders.

## 3 Results

### 3.1 Participant characteristics

**Figure 1:**
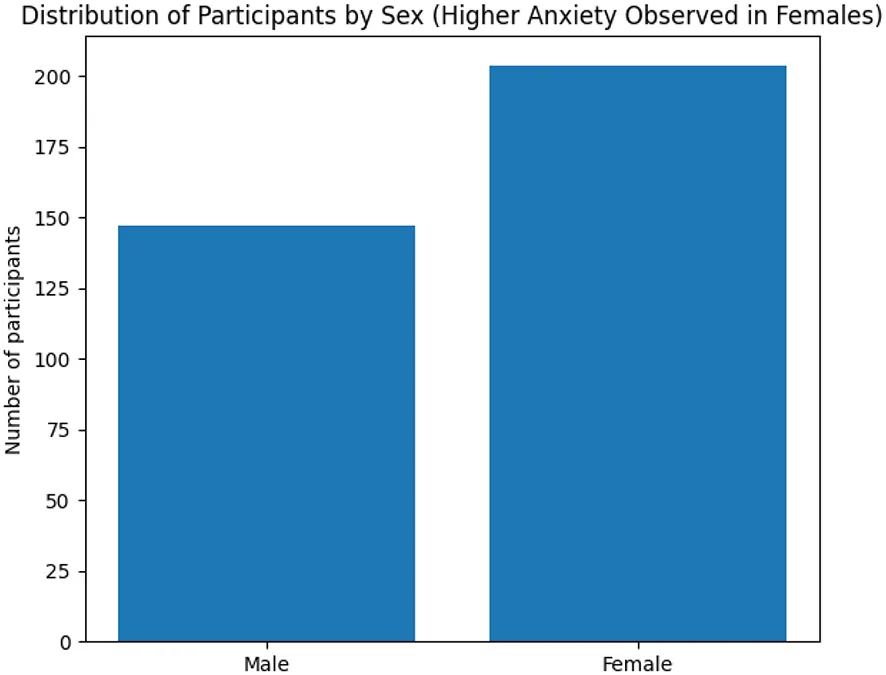
Distribution of study participants by sex (female vs. male). This figure depicts sample composition only; sex-related differences in preprocedural anxiety scores (Section 3.3) are not derived from this figure.

A total of 351 patients were included in the study. The mean age was 53.2 years (SD 15.3), with a predominance of women (58.1%) compared to men (41.9%; Figure 1 and Table 1).

**Table 1:** Sociodemographic characteristics of the study population.

Variable	Value
Total participants	351
15.6-7.2,-1.3242ptAge, mean (SD)	53.2 (15.3)
Sex, *n* (%)	
Female	204 (58.1%)
Male	147 (41.9%)

SD, standard deviation.

**Figure 2:**
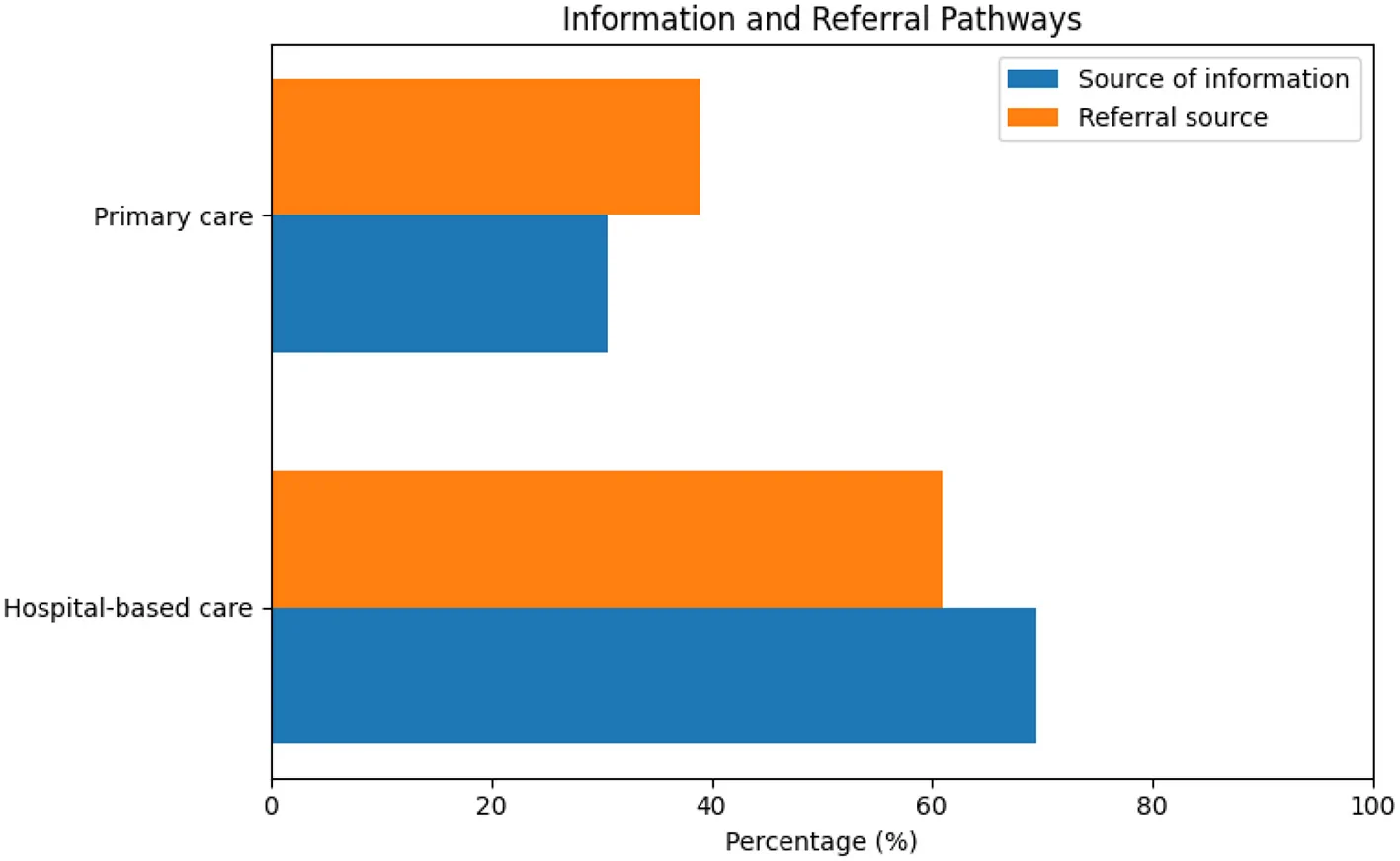
Distribution of source of information and referral pathways among patients undergoing gastrointestinal endoscopy with sedation.

Regarding information pathways, most patients reported receiving preprocedural information in the hospital setting (69.5%; *n* = 244), while 30.5% (*n* = 107) reported receiving it in primary care. Referrals were more frequently made by hospital specialists (61.0%; *n* = 214) than by primary care physicians (39.0%; *n* = 137; Figure 2).

### 3.2 Preprocedural anxiety levels and information needs

**Figure 3:**
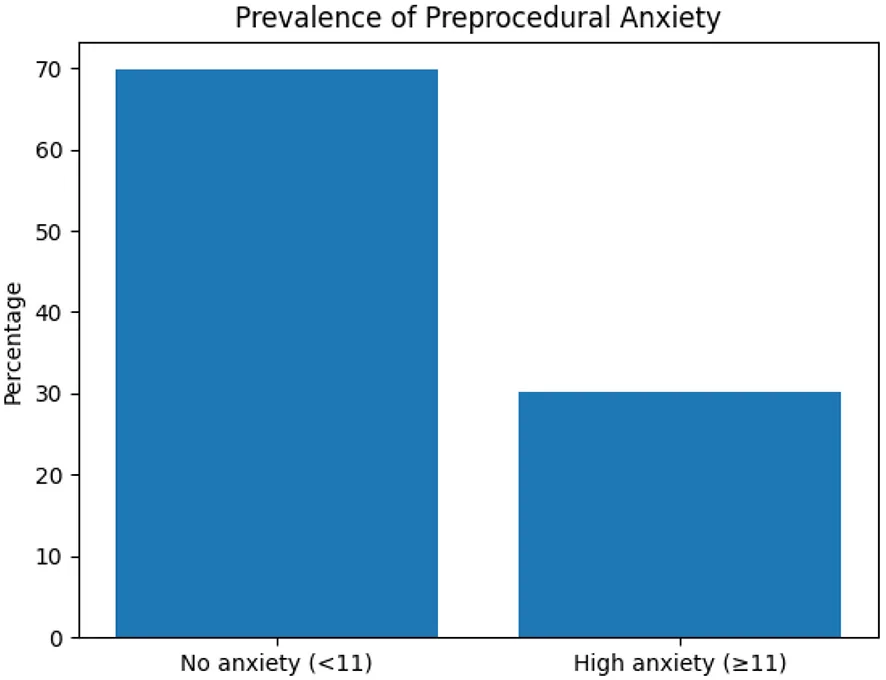
Prevalence of clinically significant preprocedural anxiety (APAIS ≥ 11) among patients undergoing gastrointestinal endoscopy with sedation.

The mean APAIS anxiety subscale score was 8.77 (SD 4.80; range 4–20). Overall, 30.2% of patients (*n* = 106) presented clinically significant anxiety (anxiety subscale ≥11), indicating that nearly one in three patients experienced elevated anxiety prior to the procedure (Figure 3).

Regarding information needs, 34.0% of patients (*n* = 119) showed high information needs (APAIS information subscale ≥8), indicating that approximately one in three patients also perceived an unmet need for further information about the procedure (Table 2).

**Table 2:** Preprocedural anxiety levels assessed using APAIS.

Variable	Value
APAIS anxiety subscale score, mean (SD)	8.77 (4.80)
Clinically significant anxiety (anxiety subscale ≥11), *n* (%)	106 (30.2)
High information needs (information subscale ≥8), *n* (%)	119 (34.0)

APAIS, Amsterdam Preoperative Anxiety and Information Scale; SD, standard deviation.

Anxiety subscale: items 1, 2, 4, 5 (range 4–20). Information subscale: items 3, 6 (range 2–10).

### 3.3 Differences according to sex and age

Significant differences in anxiety levels were observed according to sex. Women showed higher APAIS scores compared to men, and this difference was statistically significant (*p* < 0.001).

In contrast, no significant association was found between age and anxiety levels, suggesting that preprocedural anxiety is not strongly influenced by age in this population.

No significant differences were identified in information needs between men and women (*p* = 0.233).

### 3.4 Source of information and anxiety

When comparing anxiety levels according to the healthcare professional providing preprocedural information, patients informed in primary care (*n* = 107) showed a slightly higher mean total APAIS score (15.51 ± 6.93) compared to those informed by hospital-based professionals (*n* = 244; mean total APAIS 14.58 ± 6.82). However, this difference did not reach statistical significance (*p* = 0.220; Table 3).

**Table 3:** Source of information and referral pathways.

Variable	*n* (%)
Source of information	
Hospital-based care	244 (69.5%)
Primary care	107 (30.5%)
Referral source	
Hospital specialist	214 (61.0%)
Primary care physician	137 (39.0%)

### 3.5 Association between information and anxiety

The analysis of the relationship between the adequacy of information and anxiety levels revealed that a lack of explanation about the procedure was significantly associated with increased anxiety (PR 1.61; 95% CI 1.14-2.25; *p* = 0.0074).

**Figure 4:**
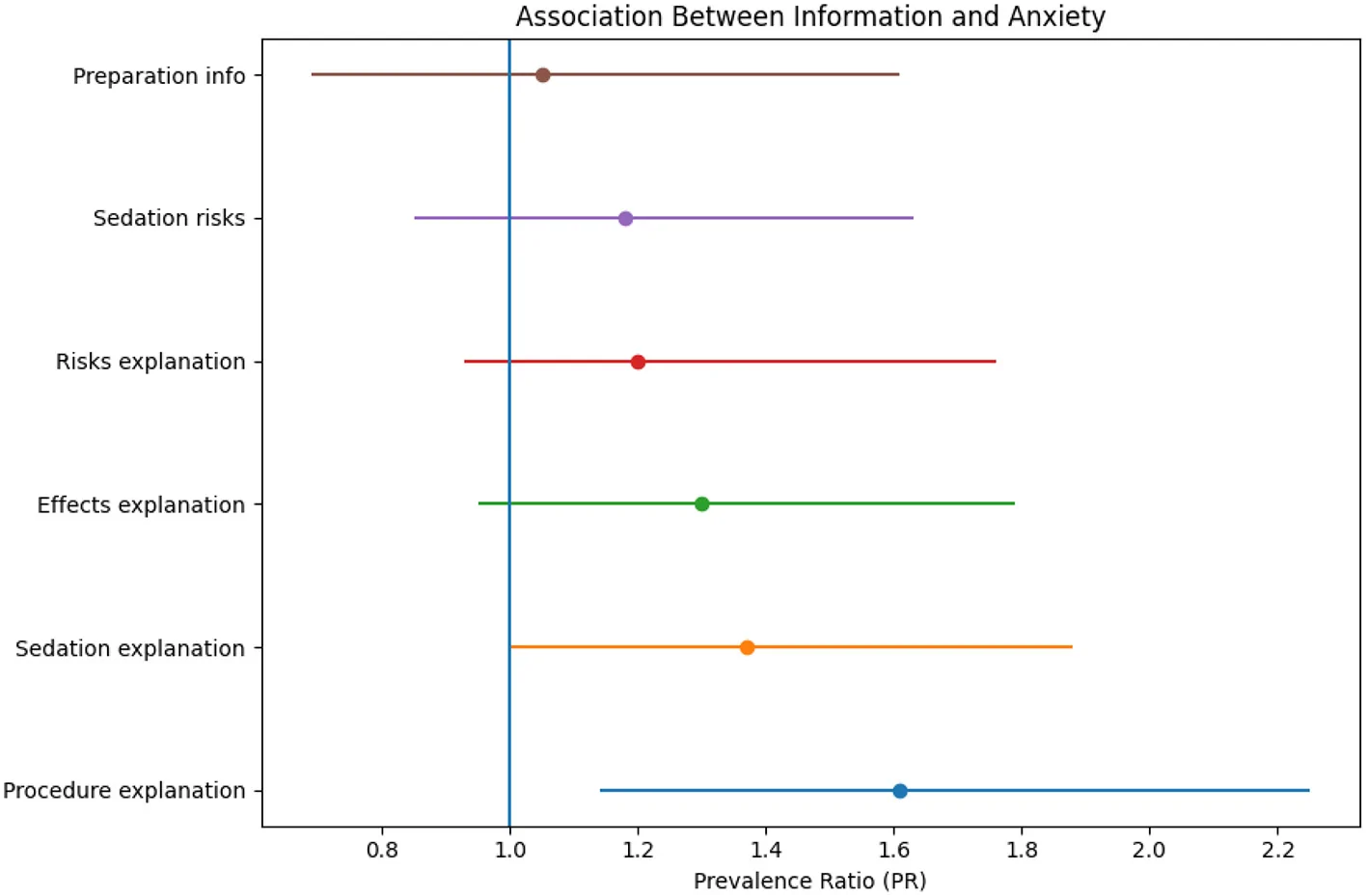
Association between lack of preprocedural information and preprocedural anxiety. Data are presented as prevalence ratios (PR) with 95% confidence intervals.

Although not reaching statistical significance, the lack of explanation regarding sedation showed a trend toward increased anxiety (PR 1.37; 95% CI 1.00-1.88; *p* = 0.0525). Similarly, insufficient information about the effects and risks of the procedure showed non-significant trends toward higher anxiety levels (Figure 4).

No significant associations were found between anxiety and information about preparation (*p* = 0.8042) or sedation risks (*p* = 0.3005).

Overall, these findings suggest that preprocedural anxiety is prevalent and primarily associated with modifiable factors related to the adequacy of information provided to patients (Table 4).

**Table 4:** Association between adequacy of information and preprocedural anxiety.

Variable	PR	95% CI	*p*-value
Lack of explanation of procedure	1.61	1.14–2.25	0.0074
Lack of explanation of effects	1.30	0.95–1.79	0.0995
Lack of explanation of risks of procedure	1.20	0.93–1.76	0.1282
Lack of explanation of sedation	1.37	1.00–1.88	0.0525
Lack of explanation of sedation risks	1.18	0.85–1.63	0.3005
Lack of preparation information	1.05	0.69–1.61	0.8042

PR, prevalence ratio; CI, confidence interval. Statistical significance set at *p* < 0.05.

## 4 Discussion

This study provides real-world evidence that preprocedural anxiety is a highly prevalent issue among patients undergoing gastrointestinal endoscopy with sedation, affecting approximately one-third of the study population. These findings are consistent with previous literature reporting substantial levels of anxiety in similar clinical settings, despite advances in sedation techniques and patient care (6, 8, 14).

One of the most relevant findings of this study is the significant association between inadequate procedural information and increased anxiety. Specifically, the lack of explanation about the procedure emerged as the key determinant of anxiety, reinforcing the notion that uncertainty and insufficient understanding are major drivers of emotional distress in preprocedural settings. These results are in line with previous studies showing that inadequate communication is one of the main contributors to anxiety before endoscopic procedures (11, 15).

Importantly, our findings suggest that it is not the source of information, but rather its quality and completeness, that may be more relevant to anxiety levels than the setting in which it is delivered. Although patients informed in primary care showed slightly higher total APAIS scores than those informed in hospital settings, these differences were not statistically significant. This highlights that variability in information delivery across care levels may be less clinically relevant than ensuring that patients receive clear, structured, and comprehensive explanations, regardless of the setting. Another key finding is the higher anxiety level observed in women, which is consistent with a substantial body of literature indicating that women are more likely to experience preprocedural anxiety (16, 17). This may be related to differences in emotional processing, risk perception, and coping strategies. Notably, no significant sex-based differences were found in information needs, suggesting that the higher anxiety in women is not explained by greater unmet informational needs, but rather by differential emotional responses to the preprocedural context.

Interestingly, age was not significantly associated with anxiety levels in our study. This contrasts with some previous reports suggesting that younger patients may exhibit higher anxiety, although findings in this regard remain inconsistent across studies (7). These discrepancies may reflect differences in study populations, measurement instruments, or contextual factors such as prior endoscopic experience.

From a methodological perspective, the use of the APAIS scale is a notable strength. Unlike other commonly used instruments such as the State-Trait Anxiety Inventory (STAI), the Depression, Anxiety and Stress Scale (DASS), or the Hospital Anxiety and Depression Scale (HADS), APAIS is specifically designed to assess both preprocedural anxiety and information needs simultaneously, providing a more clinically relevant and context-specific evaluation (13, 18). The use of modified Poisson regression with robust variance estimation is the recommended approach for estimating prevalence ratios in cross-sectional studies with common binary outcomes, as it avoids the overestimation of associations inherent to logistic regression in this context.

These findings are consistent with randomized evidence demonstrating that structured patient education before colonoscopy improves bowel preparation quality and patient-centered outcomes. Notably, Hattab et al. recently showed in a randomized controlled trial that a multimodal educational intervention significantly increased adequate bowel preparation rates compared with standard instructions, also assessing anxiety and satisfaction outcomes (19). The present study extends this evidence to the psychological domain: insufficient procedural explanation was associated with higher preprocedural anxiety, suggesting that the benefits of structured patient education may extend beyond technical preparation to encompass emotional outcomes. Taken together, these findings reinforce the need for systematic, patient-centered education as a standard component of the pre-endoscopy pathway.

The finding that 34.0% of patients also reported high information needs (APAIS information subscale ≥8) is noteworthy, particularly given that this proportion exceeds the prevalence of clinically significant anxiety (30.2%). This divergence suggests that anxiety management and information provision are complementary but distinct priorities in pre-endoscopy care.

### 4.1 Clinical implications

The findings of this study have direct implications for clinical practice. Given that inadequate information was identified as a key modifiable factor associated with anxiety, implementing standardized preprocedural education protocols should be considered a priority in endoscopy units.

These protocols should include clear explanations of the procedure, sedation, potential risks, and expected outcomes, delivered in a patient-centered manner. Additionally, incorporating multimodal strategies—such as audiovisual materials, digital platforms, or structured educational interventions—may further enhance patient understanding and reduce anxiety.

Particular attention should be given to high-risk groups, such as women and patients who report unmet informational needs, as they may benefit the most from targeted interventions.

### 4.2 Limitations

This study has several limitations that should be acknowledged. First, it was conducted in a single tertiary center, which may limit the generalizability of the findings to other settings with different patient populations, organizational models, or information-delivery practices. Second, anxiety was assessed at a single time point immediately prior to the procedure; temporal variations in anxiety levels—including post-procedural changes—could not be evaluated. Third, the adequacy of preprocedural information was determined through patient self-report, which is subject to recall bias and individual perception bias: patients may differ in what they consider adequate explanation, independent of the actual content delivered. Fourth, the APAIS, while validated, is a self-reported scale and may underestimate anxiety in contexts where social desirability influences responses. Fifth, the analysis was limited to unadjusted prevalence ratios. Potentially relevant confounders—including prior endoscopic experience, indication for the procedure, psychiatric history, health literacy, and education level—were not collected and could not be controlled for. Sixth, no procedural outcome data were collected (e.g., Boston Bowel Preparation Scale score, sedation dose, procedure tolerance, completion rate, patient cooperation, or post-procedural satisfaction). The inclusion of such outcomes would have allowed assessment of whether preprocedural anxiety or information adequacy translate into differences in clinical performance, and their absence represents a relevant limitation of the current study. Finally, the cross-sectional observational design precludes causal inferences: the reported associations between information adequacy and anxiety should be interpreted as hypothesis-generating rather than definitive evidence of a causal relationship.

### 4.3 Future directions

Although randomized evidence already supports the value of structured patient education before colonoscopy for improving bowel preparation and patient experience (19), anxiety-specific outcomes remain secondary endpoints in most existing trials. Future research should prioritize larger, multicenter, randomized studies specifically designed to evaluate structured preprocedural education as an intervention to reduce anxiety as a primary outcome. Emerging approaches such as virtual reality, mobile health applications, and interactive digital tools have shown promising results in endoscopic settings (20, 21) and warrant further evaluation in anxiety-focused trials. Longitudinal studies are also needed to determine whether reductions in preprocedural anxiety translate into measurable improvements in procedural outcomes such as tolerance, sedation requirements, and patient satisfaction.

## 5 Conclusions

Preprocedural anxiety affects approximately one in three patients undergoing gastrointestinal endoscopy with sedation, and is significantly associated with insufficient explanation of the procedure. A similar proportion of patients reports high information needs, highlighting that anxiety management and information provision are complementary but distinct priorities in pre-endoscopy care. Women constitute a particularly vulnerable group for anxiety. These findings support the priority implementation of structured, patient-centered educational strategies in endoscopy units.

## Data Availability

The original contributions presented in the study are included in the article/supplementary material, further inquiries can be directed to the corresponding author.
